# Cetuximab-Conjugated Magnetic Poly(Lactic-co-Glycolic Acid) Nanoparticles for Dual-Targeted Delivery of Irinotecan in Glioma Treatment

**DOI:** 10.3390/ma16165526

**Published:** 2023-08-08

**Authors:** Banendu Sunder Dash, Yu-Jen Lu, Shu-Hui Luo, Jyh-Ping Chen

**Affiliations:** 1 Department of Chemical and Materials and Materials Engineering, Chang Gung University, Kwei-San, Taoyuan 33302, Taiwan; banendusunder@gmail.com (B.S.D.);; 2 Department of Neurosurgery, Chang Gung Memorial Hospital at Linkou, School of Medicine, Chang Gung University, Kwei-San, Taoyuan 33305, Taiwan; 3 Craniofacial Research Center, Chang Gung Memorial Hospital at Linkou, Kwei-San, Taoyuan 33305, Taiwan; 4 Research Center for Food and Cosmetic Safety, College of Human Ecology, Chang Gung University of Science and Technology, Kwei-San, Taoyuan 33302, Taiwan; 5 Department of Materials Engineering, Ming Chi University of Technology, Tai-Shan, New Taipei City 24301, Taiwan

**Keywords:** magnetic nanoparticles, poly(lactic-co-glycolic acid), cetuximab, irinotecan, drug delivery, cancer therapy

## Abstract

A glioma is the most common malignant primary brain tumor in adults and is categorized according to its growth potential and aggressiveness. Within gliomas, grade 4 glioblastoma remains one of the most lethal malignant solid tumors, with a median survival time less than 18 months. By encapsulating CPT-11 and oleic acid-coated magnetic nanoparticles (OMNPs) in poly(lactic-co-glycolic acid) (PLGA) nanoparticles, we first prepared PLGA@OMNP@CPT-11 nanoparticles in this study. After conjugating cetuximab (CET) with PLGA@OMNP@CPT-11, spherical PLGA@OMNP@CPT-11-CET nanoparticles with 250 nm diameter, 33% drug encapsulation efficiency, and 22% drug loading efficiency were prepared in a single emulsion/evaporation step. The nanoparticles were used for dual-targeted delivery of CPT-11 to U87 primary glioblastoma cells by actively targeting the overexpressed epidermal growth factor receptor on the surface of U87 cells, as well as by magnetic targeting. The physicochemical properties of nanoparticles were characterized in detail. CET-mediated targeting promotes intracellular uptake of nanoparticles by U87 cells, which can release four times more drug at pH 5 than at pH 7.4 to facilitate drug release in endosomes after intracellular uptake. The nanovehicle PLGA@OMNP-CET is cytocompatible and hemocompatible. After loading CPT-11, PLGA@OMNP@CPT-11-CET shows the highest cytotoxicity toward U87 compared with free CPT-11 and PLGA@OMNP@CPT-11 by providing the lowest drug concentration for half-maximal cell death (IC_50_) and the highest rate of cell apoptosis. In orthotopic brain tumor-bearing nude mice with U87 xenografts, intravenous injection of PLGA@OMNP@ CPT-11-CET followed by guidance with a magnetic field provided the best treatment efficacy with the lowest tumor-associated signal intensity from bioluminescence imaging.

## 1. Introduction

Glioblastoma multiforme (GBM) is the most aggressive type of primary brain tumor and accounts for ~70% of all malignant gliomas. This type of brain tumor has only limited treatment options. The median survival time is only less than 18 months, with a five-year survival rate of only 10% [1]. Irinotecan (CPT-11), a chemotherapeutic medication marketed as Camptosar^®^, is beneficial in the treatment of GBM by blocking the DNA eukaryotic enzyme topoisomerase I [2]. Like other chemotherapeutic drugs for cancer therapy, the most significant impediment to successful use of this drug may be the side effects, as cytotoxic drugs for cancer chemotherapy can reach almost all cells in the body and cannot distinguish between normal and malignant cells [3]. Thus, this constraint in conventional brain cancer chemotherapy demands the development of a nanomaterial-based targeted drug delivery strategy, where greater selectivity toward cancer cells could be accomplished while imposing minimal impact on normal brain tissue to prevent adverse side effects [4].

A nanomedicine approach for drug delivery can deliver chemotherapeutic agents using nanoparticles. It is a feasible way for cancer treatments with the nanoscale-size and unique properties provided by the nanoparticles, which can provide many advantages over molecular therapeutics in traditional cancer chemotherapy [5]. For controlled drug delivery, the polymeric nanoparticles can encapsulate an anticancer drug for sustained drug release after intracellular uptake [6]. Furthermore, tumor-targeting nanoparticles can be prepared for cancer treatment [7]. Polymeric nanoparticles can be prepared from synthetic or natural polymers. An extensively investigated synthetic polymer for drug delivery, the biocompatible aliphatic polyester poly(lactic-co-glycolic acid) (PLGA) has been approved by the US FDA for biomedical applications [8]. Therefore, polymeric nanoparticles made from PLGA can be used as a nanovehicle for the delivery of different therapeutic agents, such as anti-inflammatory drugs, antioxidants, antibiotics, or proteins, for treating various diseases [9]. The potential to use PLGA nanoparticles as drug carriers in treating cancers has been discussed in recent review papers [10,11].

For biological applications, inorganic nanoparticles can be co-entrapped with drugs during the preparation of PLGA nanoparticles [12]. Therefore, iron oxide magnetic nanoparticles (MNP) can be incorporated into PLGA nanoparticles to prepare drug-loaded PLGA magnetic particles for magnetic targeted drug delivery [13]. Using a permanent magnet for magnetic guidance after injection of the nanocomposites, drug delivery to a diseased site in the body can be enhanced dramatically via magnetic targeting to increase drug efficacy while reducing systemic toxicity [14]. Other than magnetic targeting, the targeting ability of drug-loaded magnetic nanoparticles can be strengthened by the conjugation of a targeting ligand with their surface [15]. This active targeting is achieved through the specific bindings between ligand-conjugated nanoparticles and cancer cells, which have ligand-recognizable receptors overexpressed on their surface, to increase the intracellular uptake rates of nanoparticles by the cancer cells and improve the therapeutic efficacy [16]. For chemotherapy, this strategy can significantly enhance the cytotoxicity of drugs toward cancer cells by increasing the intracellular drug concentration found in cancer cells while reducing adverse side effects [17].

Recently, nanoparticles have emerged as a promising approach for the treatment of brain tumors [18]. Specifically, using ligand-conjugated nanoparticles, active targeting of brain cancer cells can be accomplished through receptor-mediated endocytosis [19]. Most brain tumors overexpress the epidermal growth factor receptor (EGFR), which can be targeted by cetuximab (CET), an EGFR monoclonal antibody, for drug delivery to GBM patients [20]. Doxorubicin-loaded graphene oxide was modified with CET to improve its intracellular uptake rate by glioma cells for targeted drug delivery in glioblastoma therapy [21]. Similarly, thermosensitive magnetic liposomes were surface-conjugated with CET for targeted drug delivery of CPT-11 in the chemotherapy of brain tumors [22]. We postulate that this active targeting approach could be augmented by magnetic field-induced dual-targeted CPT-11 delivery using magnetic CET-conjugated nanoparticles to improve the treatment efficacy of brain tumors. This approach is effective in dual-targeted cancer chemotherapy using transferrin-targeted [23] or CD-44-targeted [24] PLGA magnetic nanoparticles.

**Figure 1:**
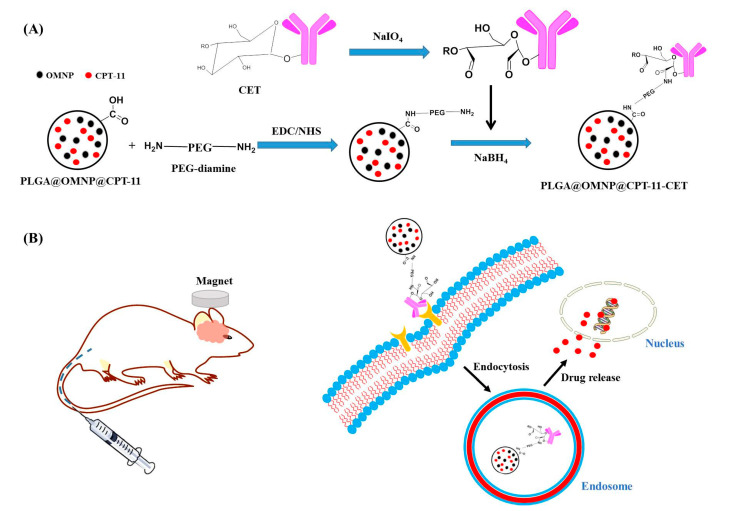
The mechanism of formation of PLGA@OMNP@CPT-11-CET (**A**) and dual-targeted glioma treatment in an orthotopic brain tumor mouse model (**B**).

Therefore, we co-entrapped CPT-11 and oleic acid-coated MNP (OMNP) in PLGA) to prepare PLGA@OMNP@CPT-11 nanoparticles by an emulsion/solvent evaporation process. The PLGA@OMNP@CPT-11 was surface-modified with PEG-diamine, leaving free amine groups on the nanoparticle surface for binding with CET, to produce PLGA@OMNP@CPT-11-CET (Figure 1A). This nanoparticle was employed for delivery of CPT-11 to U87 human primary glioblastoma cells with overexpressed EGFR on their surface. A drastic increase in cytotoxicity was shown by PLGA@OMNP@CPT-11-CET in vitro as a result of pH-sensitive CPT-11 release in the endosomes after its extracellular uptake by cancer cells. The enhanced death of cancer cells under the influence of a magnetic field in vitro is shown for magnetic targeting. The enhanced treatment for GBM is demonstrated in an orthotopic brain tumor with an intracranial U87 xenograft mouse model, where dual targeting reveals the best outcomes for GBM treatment in vivo (Figure 1B).

## 2. Materials and Methods

### 2.1. Materials

Poly(lactic-co-glycolic acid) (PLGA) with 50% lactide and 50% glycolide (intrinsic viscosity = 0.45 dL/g, molecular weight = 15,000 to 30,000 g/mole) was purchased from Green Square Co., (Taipei, Taiwan). Oleic acid (OA), 5(6)-carboxyfluorescein N-hydroxysuccinimide ester (NHS-fluorescein), and polyvinyl alcohol (PVA) (hydrolyzed) were procured from Sigma-Aldrich (St Louis, MO, USA). Poly(ethylene glycol) diamine HCl salt (PEG diamine, H_2_N-PEG-NH_2_, molecular weight = 3500) was purchased from JenKem Company (Plano, TX, USA). A Pierce™ BCA protein assay kit, Dulbecco’s modified Eagle’s medium (DMEM), and fetal bovine serum (FBS) were obtained from Thermo Fisher Scientific (Waltham, MA, USA). Life Technologies (Carlsbad, CA, USA) supplied the dyes, phalloidin–tetramethylrhodamine B isothiocyanate (phalloidin-TRITC) and 4′,6-diamidino-2-phenylindole (DAPI), for staining the actin cytoskeleton and nucleus, respectively. A CellTiter 96^®^ AQueous One Solution Cell Proliferation Assay kit for MTS assays was obtained from Promega (Madison, WI, USA).

### 2.2. Preparation of Drug-Loaded PLGA Magnetic Nanoparticles (PLGA@OMNP@CPT-11)

The preparation of PLGA magnetic nanoparticles (PLGA@OMNP) followed a previous method developed by us [24]. Iron oxide magnetic nanoparticles (MNP) were synthesized by a co-precipitation method by dissolving FeCl_3_.6H_2_O (2.15 g) and FeCl_2_.4H_2_O (0.79 g) in 30 mL deionized (DI) water. After heating to 60 °C, a flask was purged under nitrogen for 10 min and stirred vigorously. After adding 5 mL 37% (*w*/*w*) NH_4_OH and continued stirring for 30 min, the iron oxide MNP (IOMNP) nanoparticles were washed with 50 mL DI water. The pH value of the solution was lowered to 5, and the solution was sonicated and heated to 60 °C under stirring. After adding 10 mL of OA solution, which was prepared in acetone at a weight percentage of 30%, the suspension was reacted for 30 min. After collecting OA-coated MNP by magnetic separation, the OMNP was dispersed in chloroform and stored at 4 °C. The PLGA@OMNP@CPT-11 was synthesized with an emulsion/solvent evaporation process. After mixing 2.5 mL of acetone containing 50 mg PLGA and 0.25 mL of DCM, 0.5 mL OMNP (10 mg/mL) in chloroform was added and the solution was sonicated for 30 s to disperse OMNP. To create a W/O emulsion, 4 mg of CPT-11 dissolved in 1 mL 1% (*w*/*v*) polyvinyl alcohol (PVA) aqueous solution was added to the oil phase solution prepared above and sonicated for 30 s. The mixed solution was added dropwise to 12 mL 0.3% (*w*/*v*) PVA aqueous solution and sonicated for 1 min. After mixing with 50 mL PVA aqueous solution as before, the solution was stirred at 500 rpm at 30 °C overnight to remove the organic solvents. The formed PLGA@OMNP@CPT-11 nanoparticles were recovered and washed with 10 mL PBS 3 times for storage in 10 mL DI water. The PLGA@OMNP nanoparticles were prepared similarly without adding CPT-11.

### 2.3. Preparation of CET-Conjugated PLGA@OMNP@CPT-11 (PLGA@OMNP@CPT-11-CET)

The PLGA@OMNP@CPT-11 nanocomposite was conjugated with CET following a procedure reported before [25]. By binding the –COOH groups of PLGA with one –NH_2_ terminal group of PEG-diamine through carbodiimide-mediated covalent binding formation, the –NH_2_ group introduced to –NH_2_ reacted spontaneously with aldehyde groups in CET. The aldehyde groups are generated by the reduction of the glucose sugar unit from the carbohydrates in the heavy-chain constant region (Fc) of CET antibody, leaving the variable region (Fab) to react with the EGFR on cancer cell surfaces [26]. To start the preparation, 0.5 mL PLGA@OMNP@CPT-11 (1 mg/mL) was mixed with 0.25 mL EDC/NHS (1 mg/mL/EDC and 0.25 mL 1 mg/mL NHS) prepared in PBS (pH = 7.4) and reacted in the dark for 1 h. A strong magnet was used to separate the magnetic nanoparticles, which were washed twice with distilled deionized water (ddH_2_O). The polyethylene glycol diamine (PEG-diamine) was added to the solution and reacted at room temperature for 3 h and the PLGA@OMNP@CPT-11-NH_2_ nanoparticles were washed twice with ddH_2_O. To reduce the glucose units, 0.8 mL CET (5 mg/mL) was diluted with 0.2 mL ddH_2_O and added dropwise to 0.2 mL of freshly prepared 0.1 M NaIO_4_ solution in the dark. The solution was stirred for 20 min to produce –CHO groups in CET. The activated CET was purified by passing through a PV-10 desalting column and diluted with 40 μL 0.2 M sodium carbonate buffer (pH 9.5). The activated CET was reacted with PLGA@OMNP@CPT-11-NH_2_ prepared as above for 10 min and mixed with 50 μL of freshly prepared NaBH_4_ (4 mg/mL) at 4 °C for 1 h for stabilization of the covalent bonds. Finally, a strong magnet was used for magnetic separation of PLGA@OMNP@CPT-11-CET and washed twice with ddH_2_O. The supernatant was collected and analyzed for CET protein concentration using the BCA protein assay kit.

### 2.4. Physicochemical Characterization

The nanoparticles were analyzed for particle size and zeta potential using a Nano ZS 90 Zetasizer (Malvern Instruments, Malvern, UK). The particle morphology was obtained from a JEM-1230 transmission electron microscope (TEM) from JEOL (Tokyo, Japan). For Fourier-transformed infrared (FTIR) spectroscopy analysis, a Bruker Tensor 27 spectrophotometer was used after blending the sample with KBr. From the X-ray diffraction (XRD) analysis, we used a D2 PHASER X-ray diffractometer from Bruker (Billerica, MA, USA) to measure the crystalline structure of nanoparticles. A Q50 thermogravimetric analyzer from TA Instruments (New Castle, DE, USA) was used to measure the thermal degradation properties from the thermogravimetric analysis (TGA) under nitrogen purging. The iron oxide content in nanoparticles was measured with a 710-ES inductively coupled plasma optical emission spectrometer (ICP-OES) from Varian (Palo Alto, CA, USA) after digesting the sample with 36.5% HCl before measurement. The magnetization behavior of nanoparticles was analyzed with an MPMS-3 superconducting quantum interference device (SQUID) from Quantum Design (San Diego, CA, USA) from −10,000 to 10,000 Oe at 25 °C. The nanoparticles were observed by a XE-70 atomic force microscope (Park Systems, Suwon, South Korea) after diluting the samples in alcohol and sonicated. After depositing the samples onto a freshly cleaved mica substrate, the measurements were performed in a tapping mode at room temperature in a dry state.

### 2.5. Drug Loading and Release

To determine the amount of CPT-11 encapsulated in PLGA@OMNP-CPT-11, all supernatant during the preparation of PLGA@OMNP@CPT-11 was collected and the concentration of unencapsulated CPT-11 was determined by high-performance liquid chromatography (HPLC) at 370 nm wavelength. After dilution with PBS, the sample was injected into a C18 reversed-phase column. The mobile phase was 75% 0.01 M pH 4 phosphate buffer and 25% acetonitrile and the flow rate was 1 mL/min. The amount of encapsulated CPT-11 was calculated from mass balance and the loading efficiency (LE) and encapsulation efficiency (EE) of CPT-11 were calculated: encapsulation efficiency (%) = weight of encapsulated CPT-11 (mg)/weight of initial CPT-11 (mg) × 100; loading efficiency (%) = weight of encapsulated CPT-11 (mg)/weight of magnetic PLGA nanoparticles (mg) × 100.

The release of PCT-11 from PLGA@OMNP-CPT-11-CET was determined in pH 5 or 7.4 PBS. The solution (1 mL) was incubated at 37 °C in a shaking incubator (120 rpm). The supernatant was removed completely at a specific time after collecting the nanoparticles with a magnet, after which 1 mL of PBS (pH 5 or pH 7.4) was added. The CPT-11 concentration in the release solution was determined as before. The drug release from PLGA@OMNP-CPT-11-CET was calculated as the cumulative weight of released CPT-11 (mg)/initial weight of CPT-11 (mg) × 100.

### 2.6. In Vitro Cell Culture

#### 2.6.1. Cell Culture and Intracellular Uptake

The U87 human primary glioblastoma cell line genetically modified with firefly luciferase gene was used in all vitro and in vivo studies. The cell culture medium was 90% DMEM supplemented with 10% FBS. The in vitro cell culture was carried out at 37 °C in a humidified 5% CO_2_ incubator. For intracellular uptake mediated by CET, 1 × 10^5^ U87 cells were dropped into a 22 mm cover glass in a well of a cell culture plate. The cells were cultured for 24 h for attachment and 0.1 mL DMEM medium containing fluorescein-labeled PLGA@OMNP and PLGA@OMNP-CET (200 μg/mL) was added to each well and co-cultured with U87 cells for 4 h. The cells were washed with PBS, fixed with paraformaldehyde, and incubated in Triton X-100. The permeabilized sample was stained with phalloidin–TRITC for actin cytoskeleton and DAPI for cell nucleus and observed using a LSM 510 Meta confocal laser scanning microscope (Zeiss, Wetzlar, Germany). The excitation wavelength was 340/492/577 nm (blue/green/red) and the emission wavelength 488/517/590 nm (blue/green/red).

#### 2.6.2. Cytotoxicity

For cytotoxicity induced by PLGA@OMNP-CET to normal cells, nanoparticles prepared in cell culture medium were added to 5 × 10^3^ 3T3 fibroblasts in each well of a 96-well cell culture plate to reach a final particle concentration between 0.01 and 100 μg/mL. After culturing for 24 h, the cell viability was determined using the MTS assay by reading the solution absorbance with a microplate reader at 570 nm. To assess cytotoxicity toward cancer cells, 2.5 × 10^3^ cells U87 cells were seeded in each well of a 96-well cell culture plate and cultured in a cell culture medium containing CPT-11, PLGA@OMNP@CPT-11, or PLGA@OMNP@CPT-11-CET at different drug dosage for 72 h. The cell viability was assessed using MTS assays and a four-parameter logistic function was used to calculate the drug doses that achieved 50% cytotoxicity (IC_50_).

#### 2.6.3. Live/Dead Assays and Cell Apoptosis

Cells (5 × 10^4^ U87) were cultured in each well of a 24-well plate for 24 h. A strong permanent magnet (diameter = 7.5 mm) had been glued to the center of the well for magnetic guidance. After adding PLGA@OMNP@CPT-11-CET to the cells to reach 0.001 mg/mL CPT-11, the cells were co-cultured with magnetically targeted nanoparticles for 24 h, washed with PBS, stained with a Live/Dead cell viability/cytotoxicity kit, and examined under an inverted fluorescence microscope.

Flow cytometry and Western blot analysis were employed to assess cell apoptosis. First, 2.5 × 10^6^ U87 cells in a T-75 flask were treated with CPT-11, PLGA@OMNP@CPT-11, or PLGA@OMNP@CPT-11-CET (1 µg/mL of CPT-11) for 24 h. The cells were subjected to flow cytometry analysis by incubating with annexin V–FITC and propidium iodide (PI) and analyzed with an Attune NxT flow cytometer from Thermo Fisher Scientific (Waltham, MA, USA). For Western blot analysis, a standard Western blot protocol was used after blotting with primary antibodies for phosphorylated extracellular signal-regulated kinase (pERK) and caspase 3. Images were captured using a gel imaging system after incubation with secondary antibody and peroxidase substrate solutions.

### 2.7. Xenograft Tumor Model in Nude Mice

All animal experiments were carried out following the Helsinki Declaration Guidelines and approved by Chang Gung University’s Institutional Animal Care and Use Committee. Female BALB/c nude mice were used to establish the U87 orthotopic xenograft tumor model. After injecting 3 µL of cell suspension containing 3 × 10^5^ U87 cells into the right intracranial area to a depth of 3 mm, the mice were randomized into four groups (n = 3 each) eight days after U87 implantation and injected intravenously into the tail vein with 200 μL samples. The groups included normal saline (control), CPT-11(5 mg/kg CPT-11), PLGA@OMNP-CET (5 mg/kg CPT-11), PLGA@OMNP@CPT-11 (5 mg/kg CPT-11), and PLGA@OMNP@CPT-11-CET + MF. The PLGA@OMNP@CPT-11-CET + MF group had magnetic field guidance with a rectangle-shaped magnet placed near the tumor site for 30 min post-treatment. For antitumor efficacy, bioluminescence imaging (BLI) was performed using a Xenogen IVIS-200 in vivo imaging system (IVIS) on day 21 by intraperitoneal injection of 150 µL of D-luciferin solution on the day of tumor size examination. The total bioluminescence signal intensity in the tumors from BLI was standardized by dividing the intensity with that on day 8 when the mice were randomly assigned. To visualize tumors in mice brains, a Magnetom Trio 3.0 Tesla scanner (Siemens, Munich, Germany) was used for magnetic resonance imaging (MRI) on day 21. To perform the biodistribution study, 100 μL of fluorescein-labeled PLGA@OMNP-CET was injected into the tail veins of tumor-bearing mice with or without magnetic field guidance. Four hours after administration, the mice were sacrificed and organs (kidney, spleen, liver, lungs and heart) were harvested to determine the accumulation of nanoparticles in each organ using IVIS. The distribution of nanoparticles to each organ was calculated from the fluorescence intensity in each organ (n = 3).

### 2.8. Statistical Analysis

All data were repeated in triplicates and recorded as mean ± standard deviation (mean ± SD). The statistical method used single-factor analysis of variance (ANOVA) to compare the differences between each group, and *p* < 0.05 was defined as statistically significant.

## 3. Results

### 3.1. Preparation and Characterization of Nanoparticles

**Figure 2:**
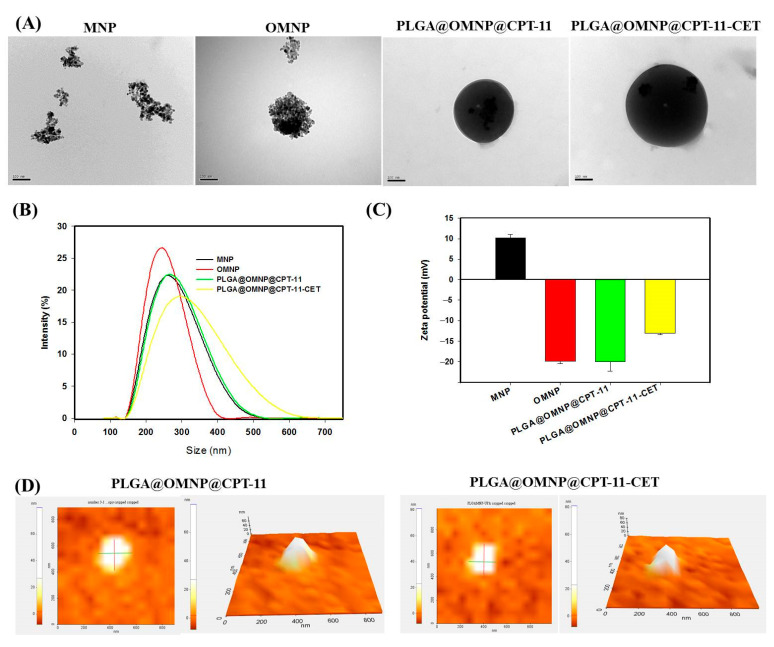
Characterization of nanoparticles by a transmission electron microscope (TEM) (bar = 100 nm) ((**A**), bar = 100 nm), dynamic light scattering (DLS) (**B**), zeta potential measurements (**C**), and atomic force microscopy (**D**).

The nanoparticles were synthesized by entrapping OMNP in PLGA. The use of OMNP is necessary for stably suspending them in the organic phase during the emulsification step. From TEM, both MNP and OMNP are prone to form aggregates in water (Figure 2A). However, discrete nanoparticles with sizes between 10 and 20 nm can still be observed. The van der Waals or dipole–dipole force between nanoparticles can cause aggregation [27]. Previously, co-precipitation methods for iron oxide MNP synthesis also produced nanoparticles showing similar aggregation behavior in an aqueous solution [28]. After co-encapsulating the CPT-11 and OMNP in the PLGA, the PLGA@OMNP@CPT-11 showed spherical particulate morphology with smooth contours. The particles were black with OMNP distributed throughout the interior of the PLGA nanoparticles, indicating the OMNP had been well encapsulated within the polymeric matrix. After surface modification with CET, there was little change in the surface morphology of PLGA@OMNP@CPT-11-CET from that of PLGA@OMNP@CPT-11 from the TEM image. All nanoparticles showed one peak in the DLS results (Figure 2B). The TEM data were further supported by the average particle size obtained from DLS analysis (Table 1). In general, the particles were larger than that reported for paclitaxel-loaded magnetic PLGA nanoparticles (130 nm) [23], or doxorubicin-loaded (80 nm) magnetic PLGA nanoparticles [29]. The polydispersity index (PDI) values from DLS were all below 0.3, indicating the unimodal size of nanoparticles. The surface potential of different samples is shown in Figure 2C and Table 1. The average zeta potential of MNP was positive at 16.2 mV due to the presence of residual ammonium ions after synthesis. After coating with OA, the zeta potential changed to −19.8 mV from carboxylate groups in OA. The PLGA@OMNP@CPT-11 showed a negative potential at −20.6 mV from the carboxylate end group in PLGA. After conjugation of CPT-11, this value shifted to the positive side and became −13.0 mV, as CET will carry a positive charge at a neutral pH value with its isoelectric point (pI) being 8.5 [26]. The amount of CET in PLGA@OMNP@CPT-11-CET was calculated to be 83 μg/mg particle by determining the CET concentration from the BCA protein assay. This can be compared with the amount of CET conjugated with magnetic liposomes (87 μg/mg) [22]. The morphology and particle size of PLGA@OMNP@CPT-11 and PLGA@OMNP@CPT-11-CET were also determined by an atomic force microscope, and showed smooth spherical morphology with ~250 nm size (Figure 2D).

**Figure 3:**
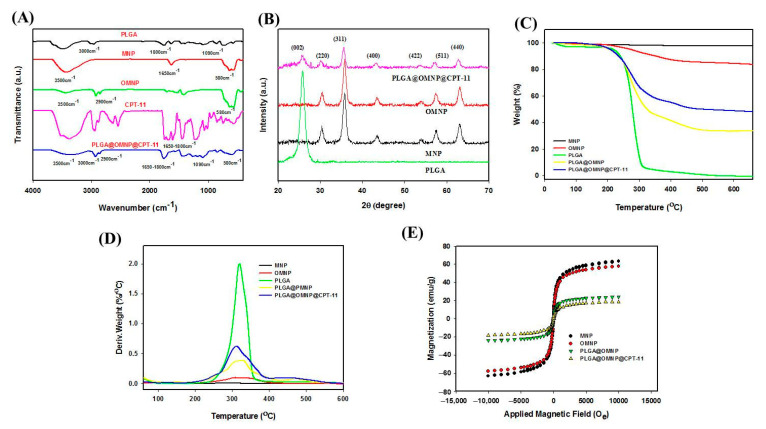
The characterization of nanoparticles by Fourier-transformed infrared (FTIR) spectroscopy (**A**), X-ray diffraction (XRD) analysis (**B**), thermogravimetric analysis (TGA) (**C**), differential thermal analysis (DTA) (**D**), and superconducting quantum interference device (SQUID) analysis (**E**).

The Fourier-transformed infrared (FTIR) spectroscopy analysis of all nanoparticles is shown in Figure 3A. The PLGA shows –C=O, –C–O–C–, and –C–H– characteristic peaks at 1800, 1090, and 3000 cm^−1^ [30]. The MNP reveals characteristic peaks at 3500 cm^−1^ (–OH), 1650 cm^−1^ (–C=O), and 580 cm^−1^ (Fe–O) [31]. A new peak appears in the spectrum of OMNP compared with MNP at 2900 cm^−1^_,_ which could be assigned to the –CH group of OA [32]. The CPT-11 has a C=O characteristic peak between 1600 and 1800 cm^−1^. The PLGA@OMNP@CPT-11 shows all characteristic peaks associated with its components, especially the –C–O–C– peak in PLGA only, but not in OA [33], endorsing the successful preparation of PLGA@OMNP@CPT-11. The XRD analysis results shown in Figure 3B indicate six characteristic peaks for all samples, corresponding to the (220), (311), (400), (422), (511), and (440) lattice planes of iron oxide MNP [34]. The magnetite crystalline sizes calculated from the strongest (311) reflection peak with the Debye–Scherrer equation were 9.5 nm and 10.8 nm for MNP and PLGA@OMNP@CPT-11, respectively, indicating successful encapsulation of OMNP using water-in-oil emulsion [35].

From TGA, the residual weight is about 98% and 85% for MNP and OMNP, respectively after complete thermal decomposition at 700 °C (Figure 3C). The 13% difference may arise from OA coating, which could be decomposed at higher temperatures in contrast to the non-decomposable nature of MNP. The PLGA shows thermal decomposition within 200 to 400 °C with no residual weight at 700 °C due to its synthetic polymer nature. A sharp decomposition peak temperature appears in the DTG curve at 310 °C, which appears in every PLGA-based nanoparticle (Figure 3D). The residual weight is 47.6% and 33.2% for PLGA@OMNP and PLGA@OMNP@CPT-11, respectively, indicating the successful encapsulation of OMNP, as only OMNP can provide residual weight from the non-decomposable MNP in the core. The ICP-OES analysis supports the TGA data by directly showing the weight percentage of iron oxide in MNP (98.7 ± 0.9%), OMNP (87.4 ± 4.0%), PLGA@OMNP (43.0 ± 2.7%), and PLGA@OMNP@CPT-11 (40.0 ± 5.4%) (n = 3). The magnetization curves from SQUID analysis show hysteresis loops passing through the origin (Figure 3E). The remnant (residual) magnetization is close to zero (<0.3 emu/g) and endorses the superparamagnetic behavior for all tested samples, as superparamagnetism behavior depends on the size of nanoparticles and only occurs when the particle size is below ~20 nm. This observation is consistent with the particle size of OMNP from TEM and XRD analyses. The saturation magnetization values are 63.2 and 57.7 emu/g for MNP and OMNP, respectively. These values decrease to 24.1, and 18.7 emu/g for PLGA@OMNP and PLGA@OMNP@CPT-11, respectively, due to the reduced OMNP weight percentage in the nanoparticles. As the reduced saturation magnetization value was assigned to the non-magnetic component (PLGA and CPT-11), the OMNP content can be calculated by dividing the saturation magnetization value by that of OMNP, which is 41.8% for PLGA@OMNP and 32.4% for PLGA@OMNP@CPT-11.

**Figure 4:**
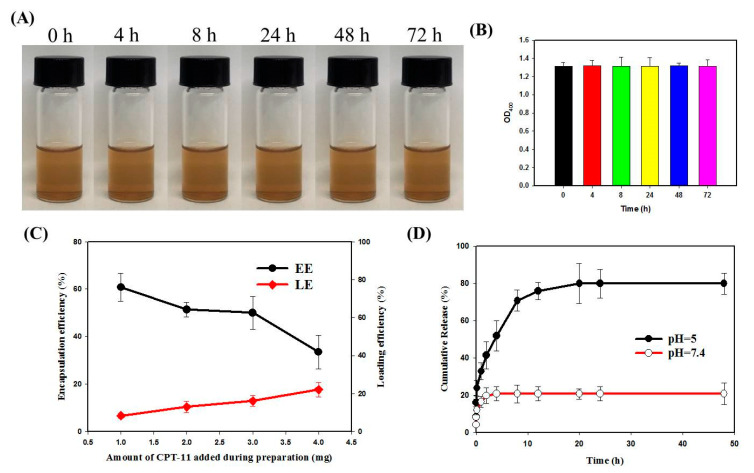
The suspension stability (**A**) and solution absorbance at 400 nm (OD_400_) of a PLGA@OMNP@CPT-11 solution (1 mg/mL) in pH 7.4 PBS (**B**). The encapsulation efficiency (EE) and the loading efficiency (LE) of CPT-11 in PLGA@OMNP@CPT-11-CET (**C**) and release profile at 37 °C in PBS (pH 5 or pH 7.4) (**D**).

The suspension stability of PLGA@OMNP@CPT-11-CET was examined in pH 7.4 PBS. As shown in Figure 4A, the nanoparticles form a well-dispersed solution without visible precipitation for up to 3 days. There is also no significant change in solution absorbance (OD_400_) (Figure 4B). Figure 4C depicts the loading efficiency (LE) and encapsulation efficiency (EE) of CPT-11. When the amount of CPT-11 utilized to prepare PLGA@OMNP@CPT-11-CET is increased from 1 to 4 mg, the LE value increases from 8.5% to 22.1%, and the EE value decreased from 60.8% to 33.5%. Because the LE could not be increased further while the EE decreased, a 4 mg CPT-11 dosage was used in all tests to create the PLGA@OMNP@CPT-11-CET. Figure 4D depicts the release of CPT-11 from the PLGA@OMNP@CPT-11-CET at 37 °C in pH 5 or 7.4 PBS. The release of CPT-11 from PLGA@OMNP@CPT-11-CET is pH-sensitive, with a faster drug release rate at pH 5 than pH 7.4. The cumulative release percentages in 48 h are 20.7% and 79.8%, respectively, at pH 7.4 and pH 5. The weakened electrostatic interaction between –NH^+^ in CPT-11 and –COOH in PLGA at a low pH value may also contribute to the difference in release rate at different pH values [36]. The faster degradation rate of PLGA in an acidic environment also contributes to the increase [37]. Overall, the close-to-fourfold increase in release rate at pH 5 indicates the acidic endosomal microenvironment can promote drug release after intracellular uptake of PLGA@OMNP@CPT-11-CET, which is expected to elicit higher cytotoxicity on U87 cancer cells, in contrast to an extracellular physiological pH value.

### 3.2. In Vitro Studies

**Figure 5:**
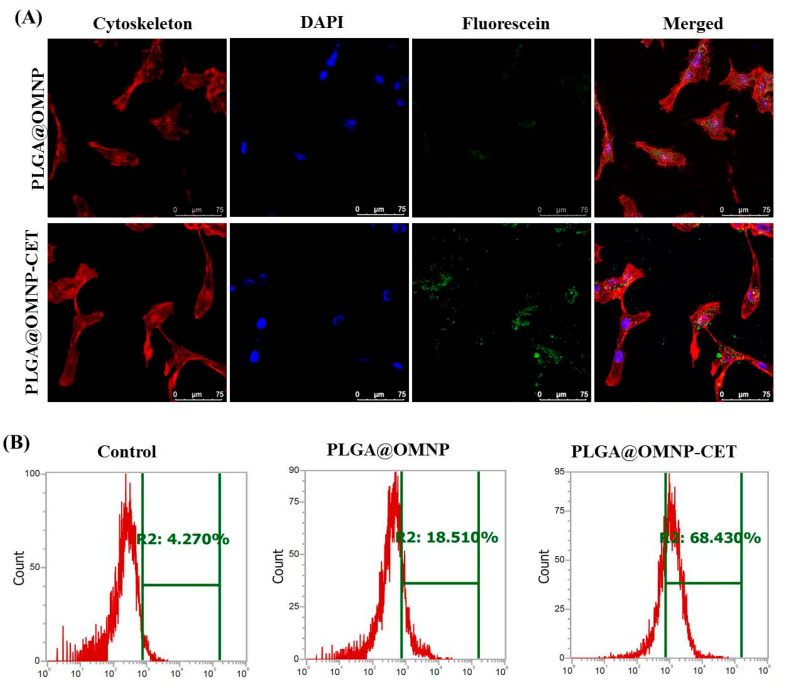
(**A**) The intracellular uptake of fluorescein-labeled PLGA@OMNP or PLGA@OMNP-CET (green fluorescence) by co-culture with U87 cells for 4 h. The cell cytoskeleton was stained with phalloidin (red fluorescence) and the nucleus was stained with DAPI (blue fluorescence). After staining, the cells were examined under a confocal laser scanning microscope (bar = 75 µm). (**B**) The flow cytometry analysis of intracellular uptake by co-culture U87 cells with PLGA@OMNP or PLGA@OMNP-CET for 4 h.

Fluorescein-labeled PLGA@OMNP and PLGA@OMNP-CET were treated with U87 cells for 4 h to determine whether CET can act as a targeting ligand toward the EGFR on the cell surface. The NHS-fluorescein (5/6-carboxyfluorescein succinimidyl ester) was directly added to the emulsion step to prepare the fluorescence-labeled nanoparticles, and the intracellular green fluorescence was detected using confocal laser scanning microscopes. The cytoskeleton was stained with phalloidin, and the nuclei were identified with DAPI. As shown in Figure 5A, the green fluorescence intensity from fluorescein bound to the nanoparticle is substantially stronger for PLGA@OMNP-CET than PLGA@OMNP, demonstrating that CET conjugation can boost the effectiveness of nanoparticle engulfment by U87. For quantitative assessment of the intracellular uptake rate, flow cytometry was used to measure the fluorescein green fluorescence signal intensity from fluorescence-activated cell sorting (FACS). The geometric mean fluorescence intensities for the control, PLGA@OMNP, and PLGA@OMNP-CET are 299, 601, and 1509, respectively, and the uptake ratio is 18.5 and 68.4% for PLGA@OMNP and PLGA@OMNP-CET, respectively (Figure 5B). By subtracting the background signal from the control, the PLGA@OMNP-CET has ~3.5-fold higher targeting efficacy than PLGA@OMNP, which is mediated by CET on the particle surface and is consistent with the results from confocal microscopy analysis. Considering conjugation of CET antibody PLAG@OMNP, we used the method for site-specific modification of an antibody through its carbohydrate moieties [38]. Thus, the carbohydrate moieties in the Fc regions of the heavy chains of CET were used for conjugation, which are not important for binding with the EGFR antigen overexpressed on the U87 surface. Overall, the intracellular uptake investigation supports the use of PLGA@OMNP-CET as a promising nanocarrier for the delivery of CPT-11 to U87 cells.

**Figure 6:**
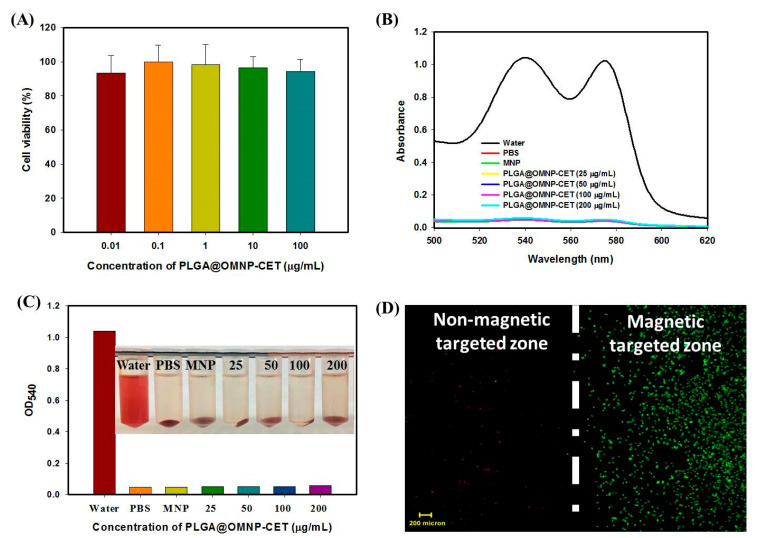
(**A**) The cytocompatibility of PLGA@OMNP-CET at different concentrations was determined from relative cell viability using the MTS assay after co-culture with 3T3 fibroblasts. (**B**) The hemocompatibility of PLGA@OMNP-CET was determined from hemolysis assays by incubating in PBS with diluted red blood cells at 37 °C for 2 h. Water was the positive control and PBS was the negative control. (**C**) The supernatant absorbance values at 540 nm (OD_540_); the inset image shows the gross view of samples. (**D**) The representative image from Live/Dead cell staining after incubating U87 with PLGA@OMNP-CPT-11-CET in a culture plate and guided with a magnetic field. The dotted line is the boundary of a magnet fixed to the bottom of a well in the culture plate.

After validating cellular internalization, the biocompatibility of nanoparticles was tested by exposing 3T3 fibroblast cells to different concentrations of PLGA@OMNP-CET for 24 h. Cell vitality was evaluated using MTS assays and standardized to that of a control cell culture medium. As shown in Figure 6A, after contacting 3T3 cell lines with 100 µg/mL PLGA@OMNP-CET in a cell culture medium for 24 h, the relative cell viability is still above 90%, indicating that the internalization of the nanocarrier exerts negligible adverse effects on cell viability.

Aside from cytocompatibility, hemocompatibility testing was carried out to determine whether nanoparticles can cause a hemolytic response when administered intravenously. To perform the in vitro hemolysis assays, different doses of PLGA@OMNP-CET were added to red blood cells withdrawn from rats after dilution and incubated for 2 h at 37 °C. As positive and negative controls, water and PBS were employed, respectively. The absorption spectra of the supernatant collected from test samples, using a scanning wavelength from 500 to 620 nm, indicate red blood cells incubated with 25 to 200 µg/mL of PLGA@OMNP-CET in PBS caused little change from that of PBS (Figure 6B). However, water leads to the rupture of red blood cells and releases oxyhemoglobin with two distinct absorption peaks at 540 and 577 nm in the positive control group. The overall appearance of all samples shows no obvious hemolysis, and no difference in OD_540_ could be found between PLGA@OMNP-CET samples and PBS (Figure 6C). PLGA@OMNP-CET demonstrates superior biocompatibility and hemocompatibility for in vivo application.

To confirm the magnetic targeting effect, we incubate PLGA@OMNP@CPT-11-CET with U87 cells in a well, which has a magnet fixed to the bottom of its surface, to create a magnetic targeted zone for guiding the nanoparticles. Using the Live/Dead cell staining, we can only find dead cells showing red fluorescence confined within the magnetic targeted zone (Figure 6D). This indicates that a magnetic field can magnetically guide the cells to enhance intracellular uptake by U87 cells and trigger cell death. In contrast, abundant live cells showing green fluorescence exist primarily outside the magnetic targeted zone, and a minimum concentration of PLGA@OMNP@CPT-11-CET is expected in the non-magnetic targeted zone (Figure 6D). An external magnetic field can therefore guide PLGA@OMNP@CPT-11-CET to the tumor location and raise local drug concentration, allowing for dual-targeted anti-cancer therapy.

**Figure 7:**
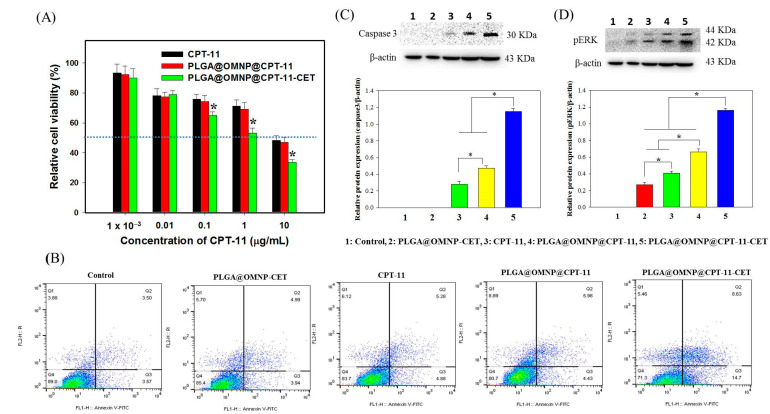
(**A**) The cytotoxicity of free and encapsulated CPT-11 towards U87 cells by incubating cells with samples of different drug dosages for 48 h. * *p* < 0.05 compared with PLGA@OMNP@CPT-11 or CPT-11. (**B**) The flow cytometry analysis of necrotic cells (Q1), late apoptotic cells (Q2), early apoptotic cells (Q3), and live cells (Q4) after different treatments for 24 h. The relative expression of the phospho-extracellular signal-regulated kinase (pERK) (**C**) and caspase 3 (**D**) was determined by Western blot analysis, and semiquantitative analysis of protein band intensity from gel images was determined using β-actin as control. * *p* < 0.05.

After confirming CET-mediated targeting and safety, the cytotoxicity against U87 cells was calculated to assess the in vitro chemotherapeutic efficacy. For this, U87 cells were treated with free or encapsulated CPT-11 at comparable drug doses at 37 °C for 48 h. Figure 7A shows that PLGA@OMNP@CPT-11-CET can elicit the most pronounced cytotoxic effect with the lowest IC_50_ (2.5 µg/mL) when compared to PLGA@OMNP@CPT-11 (8.8 µg/mL) and CPT-11 (9.3 µg/mL). The increased cytotoxicity of PLGA@OMNP@CPT-11 over CPT-11 indicates drug delivery through PLGA@OMNP can induce higher cytotoxicity toward U87 cells than using free CPT-11. This effect could be further amplified when using PLGA@OMNP@CPT-11-CET, where CET can increase the uptake rate of the nanoparticles and the intracellular CPT-11 concentration for killing the cancer cells. Next, we performed flow cytometry analysis to investigate the cell death mechanism. The proportion of necrotic (Q_1_), late apoptotic (Q_2_), early apoptotic (Q_3_), and live (Q_4_) cells was determined after staining the cells with annexin V–FITC/propidium iodide (Figure 7B). The flow cytometry analysis confirms that apoptosis is the major cell mechanism elicited by CPT-11. As demonstrated before in Figure 4A, PLGA@OMNP-CET is cytocompatible with minimum cytotoxicity. The ratio of live cells for PLGA@OMNP@CPT-11 (80.7%) is less than that for CPT-11 (83.7%), which further decreases to 71.3% for PLGA@OMNP@CPT-11-CET. To further confirm and compare the extent of cell apoptosis under different treatments, two apoptosis-related proteins, phospho-extracellular signal-regulated kinase (pERK) and caspase 3, were isolated from treated cells and subjected to Western blot analysis with β-actin as an internal control. As shown in Figure 6C,D, the levels of caspase 3 and pERK expression from semiquantitative analysis of protein band intensity in Western blot images are in the order of PLGA@OMNP-CET < CPT-11 < PLGA@OMNP@CPT-11 < PLGA@OMNP@CPT-11-CET. The apoptosis marker protein expression for PLGA@OMNP@CPT-11-CET is significantly higher than all other groups, indicating CET-mediated endocytosis can provide active targeting of PLGA@OMNP@CPT-11-CET toward U87 to induce the highest cell apoptosis rate by releasing CPT-11 [39].

### 3.3. In Vivo Studies

**Figure 8:**
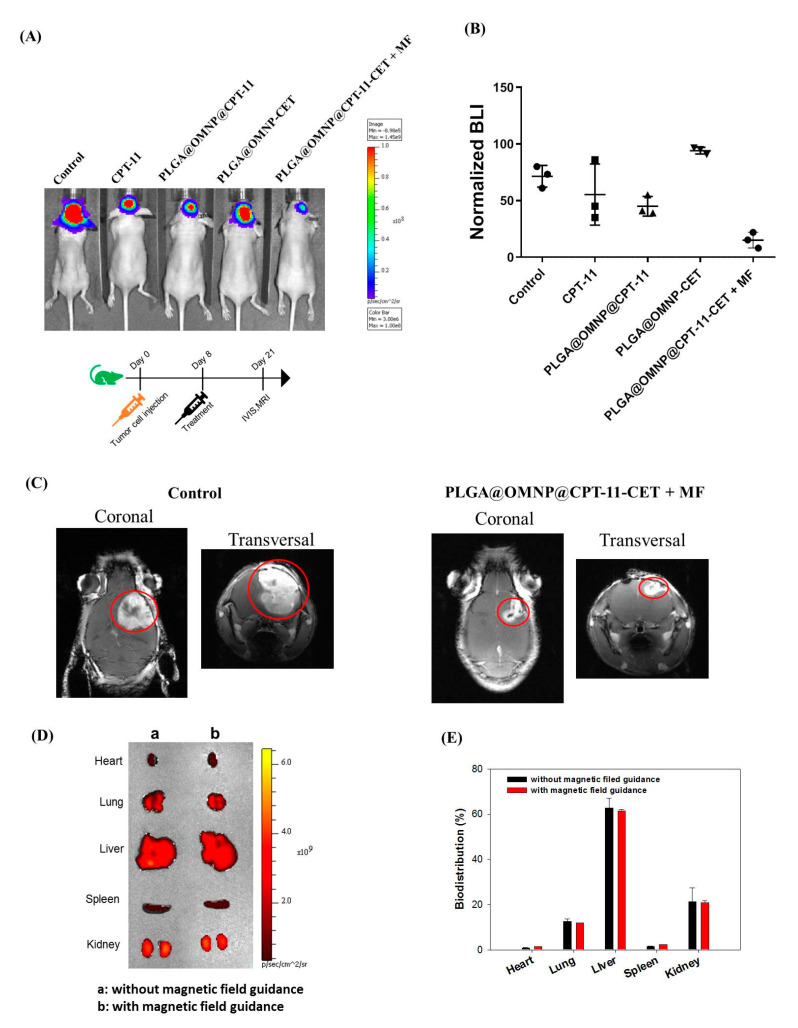
(**A**) The bioluminescence imaging (BLI) by in vivo imaging system (IVIS) on day 21 after treating U87 xenografts in nude mice bearing intracranial U87 tumors were treated with intravenous injection of different formulations. The flow diagram depicts tumor induction day, treatment start day, and treatment end day. (**B**) The normalized BLI by dividing the signal intensity on day 21 with the baseline value on day 8. (**C**) The representative coronal view and transversal view from magnetic resonance imaging (MRI) on day 21 with tumors circled in red. (**D**) The representative ex vivo fluorescence imaging of harvested organs by IVIS after administration of PLGA@OMNP-CET with or without magnetic field guidance. (**E**) The quantification of biodistribution of nanoparticles based on the fluorescence intensity in each organ.

For in vivo studies, we used an orthotopic brain tumor model from nude mice using U87 xenografts. BALB/c mice were implanted with 3 × 10^5^ U87 cells each intracranially and separated into five groups. The sample for treatment was delivered through intravenous injection of 200 μL normal saline (control) or drug solution. The drug dose selected for the animal study was based on a previous study using immunoliposomes for targeted delivery of CPT-11 and panobinostat [25]. The treatment efficacy was easily followed through bioluminescence imaging (BLI) using IVIS, as the implanted U87 cells can stably express the firefly luciferase gene. Figure 8A shows exemplary IVIS images from different groups 21 days post-implantation of U87 cells. The BLI signal intensity is highest in the control and vehicle (PLGA@OMNP-CET) groups, demonstrating that the vehicle could not elicit an anti-cancer effect in vivo and confirming the results from in vitro study. The CPT-11 group showed some decrease in BLI signal intensity from the chemotherapeutic effect and the intensity further declined when PLGA@OMNP-CPT-11-CET was used for ligand-mediated active targeting of implanted U87 cells. Most importantly, the PLGA@OMNP-CPT-11-CET + MF treatment, where dual targeting was realized through CET and magnetic field, led to the lowest bioluminescent signal in U87 xenografts. To compare treatment outcomes, the BLI signal intensity was normalized with the initial baseline value on day 8 in each group. As shown in Figure 8B, the normalized BLI is in the order of PLGA@OMNP-CPT-11-CET + MF < PLGA@OMNP-CPT-11-CET < CPT-11 < PLGA@OMNP. Most importantly, the normalized BLI of PLGA@OMNP-CPT-11-CET is significantly less than all other groups (*p* < 0.05). For brain tumor therapy, various nanoparticles as drug carriers can cross the blood–brain barrier (BBB) by different strategies, with ligand-conjugated nanoparticles showing the most promising results [40]. Specifically, PLGA nanoparticles were shown to be able to cross the BBB for the treatment of glioma [41]. Undoubtedly, dual-targeted chemotherapy with PLGA@OMNP-CPT-11-CET after induction with a magnetic field provides the best treatment efficacy. Magnetic resonance imaging (MRI) is widely used for assessing the 3D structure and size of tumors and can precisely locate tumors. To determine whether BLI can accurately reflect the size of intracranial tumors, a brain MRI was performed on U87 xenografts for the control and the best treatment group. Figure 8C shows the remarkable shrinkage in tumors (circled region) with PLGA@OMNP@CPT-11-CET + MF treatment vs. control, reconfirming the best treatment outcome is provided by dual-targeted delivery of CPT-11 with PLGA@OMNP-CPT-11-CET and guided with a magnetic field. To conduct a biodistribution study, the mice were injected with fluorescein-labeled PLGA@OMNP-CET with or without magnetic field guidance. After sacrificing the mice after 4 h, the organs were harvested to visualize the accumulation of nanoparticles in each organ by IVIS (Figure 8D). The distribution of nanoparticles among organs was calculated from the fluorescence intensity in each organ from the ex vivo images (Figure 8E). The nanoparticles were found to be mainly accumulated in the liver, with no difference between the use of magnetic field guidance or not. More than half of the administrated PLGA magnetic nanoparticles were accumulated in the liver, which is consistent with the finding that hepatic clearance represents the primary route of excretion for nanoparticles [42].

## 4. Conclusions

In this study, we successfully conjugated CET with CPT-11-loaded PLGA magnetic nanoparticles to prepare PLGA@OMNP@CPT-11-CET for anti-cancer glioma treatment using U87 cells. After modifying iron oxide MNP with OA, the OMNP can be successfully co-entrapped with CPT-11 in PLGA nanoparticles in a single emulsion/solvent evaporation step to synthesize PLGA@OMNP@CPT-11, which has a size less than 250 nm for endocytosis. By conjugating CET on the surface of the nanoparticles, enhanced intracellular uptake by U87 cells from active cell targeting was demonstrated both qualitatively from confocal microscopy and quantitatively from flow cytometry analysis. The nanoparticles can be guided by a magnetic field for magnetic targeting of U87 cells and release CPT-11 in the acidic endosomal environment for effective killing of cancer cells, and shows the highest cytotoxicity toward U87 cancer cells with the lower IC_50_ value in vitro. Using a U87 xenograft orthotopic brain tumor model, nude mice injected with PLGA@OMNP@CPT-11-CET into the tail vein followed by magnetic guidance at the tumor site showed the best treatment outcome, drastically shrinking the tumors from IVIS and MRI studies. Overall, the dual-targeting drug delivery ability associated with PLGA@OMNP@CPT-11-CET magnetic nanoparticles was shown to provide an effective nanomedicine-based chemotherapy modality for brain tumors using U87 glioblastoma cells.

## Figures and Tables

**Table 1:** The average size and polydispersity of nanoparticles were determined from dynamic light scattering (mean ± SD, n = 3).

Sample	Average Particle Size (nm)	Polydispersity Index (PDI)	Zeta Potential (mV)
MNP	239.6 ± 14.0	0.17 ± 0.04	16.2 ± 0.8
OMNP	221.3 ± 2.7	0.19 ± 0.03	−19.8 ± 0.7
PLGA@OMNP@CPT-11	237.2 ± 4.5	0.12 ± 0.02	−20.6 ± 2.3
PLGA@OMNP@CPT-11-CET	245.2 ± 5.1	0.29 ± 0.02	−13.0 ± 0.4

## Data Availability

The data presented in this study are available on request from the corresponding author.
